# Evaluation of the Aggressive-Variant Prostate Cancer Molecular Signature in Clinical Laboratory Improvement Amendments (CLIA) Environments

**DOI:** 10.3390/cancers15245843

**Published:** 2023-12-14

**Authors:** Paul V. Viscuse, Rebecca S. Slack-Tidwell, Miao Zhang, Prih Rohra, Keyi Zhu, F. Anthony San Lucas, Eric Konnick, Patrick G. Pilie, Bilal Siddiqui, Christopher J. Logothetis, Paul Corn, Sumit K. Subudhi, Colin C. Pritchard, Rama Soundararajan, Ana Aparicio

**Affiliations:** 1 Department of Medicine, University of Virginia, Charlottesville, VA 22903, USA; pviscuse@virginia.edu; 2 Department of Biostatistics, University of Texas MD Anderson Cancer Center, Houston, TX 77030, USA; 3 Department of Pathology, University of Texas MD Anderson Cancer Center, Houston, TX 77030, USAkzhu1@mdanderson.org (K.Z.); 4 Department of Hematopathology, University of Texas MD Anderson Cancer Center, Houston, TX 77030, USA; 5 Department of Laboratory Medicine and Pathology, University of Washington, Seattle, WA 98195, USA; konnick@uw.edu (E.K.);; 6 Department of Genitourinary Medical Oncology, University of Texas MD Anderson Cancer Center, Houston, TX 77030, USA; 7 Department of Translational Molecular Pathology, University of Texas MD Anderson Cancer Center, Houston, TX 77030, USA; rsoundararajan@mdanderson.org

**Keywords:** aggressive-variant prostate cancer, immunohistochemistry, molecular biomarker, next-generation sequencing

## Abstract

**Simple Summary:**

Alterations in two or more of the tumor suppressors TP53, RB1, and PTEN enrich for prostate cancers with an aggressive disease course which benefits from combination chemotherapies, but with poor survival. This signature could aid in patient selection for clinical trials that study novel therapies aimed towards these aggressive tumors. We assess the operational characteristics of staining tumor tissues, studying DNA obtained from the tumor directly, or studying DNA from tumor cells circulating in the blood to detect this signature. We encountered various challenges that limited the number of evaluable samples for all three assays in each patient. Overall, tissue staining had a higher detection rate and the shortest turnaround times, making it an attractive choice for use in clinical trials. There were operational barriers to accurately detecting the signature in tumor DNA as well as difficulty detecting sufficient tumor content in circulating tumor DNA.

**Abstract:**

*Aggressive-variant prostate cancers* (AVPCs) are a subset of metastatic castrate-resistant prostate cancers (mCRPCs) characterized by defects in ≥ two of three of *TP53*, *RB1*, and *PTEN* (AVPCm), a profile linked to lineage plasticity, androgen indifference, and platinum sensitivity. Men with mCRPC undergoing biopsies for progression were assessed for AVPCm using immunohistochemistry (IHC), next-generation sequencing (NGS) of solid tumor DNA (stDNA), and NGS of circulating tumor DNA (ctDNA) assays in CLIA-certified labs. Biopsy characteristics, turnaround times, inter-reader concordance, and inter-assay concordance were assessed. AVPCm was detected in 13 (27%) patients via IHC, two (6%) based on stDNA, and seven (39%) based on ctDNA. The concordance of the IHC reads between pathologists was variable. IHC had a higher detection rate of AVPCm^+^ tumors with the shortest turnaround times. stDNA had challenges with copy number loss detection, limiting its detection rate. ctDNA detected the greatest proportion of AVPCm^+^ tumors but had a low tumor content in two thirds of patients. These data show the operational characteristics of AVPCm detection using various assays, and inform trial design using AVPCm as a criterion for patient selection or stratification.

## 1. Introduction

A subset of prostate cancers have been dubbed ‘*androgen indifferent*’ given their poor response to existing androgen signaling inhibitors. This subset has limited therapeutic options and dismal outcomes, in part due to the absence of identifiable biomarkers [1,2,3,4,5]. To arrive at said biomarkers, we turned to the small-cell prostate carcinomas (SCPCs), a known androgen-resistant histologic variant. The SCPCs are characterized by atypical clinical features with a virulent course and sensitivity to platinum-based chemotherapy [6,7,8,9,10]. It then became apparent that some prostate adenocarcinomas shared similar features with SCPCs. We thus defined *aggressive-variant prostate cancers (AVPCs)* to encompass prostate cancers that behave like SCPCs, even when SCPC morphology is absent [11,12]. We showed that AVPC tumors are characterized by a molecular signature of combined alterations in *TP53*, *RB1*, and/or *PTEN* (AVPCm) [12].

AVPCm has been associated with lineage plasticity and androgen indifference in preclinical models [9,13,14]. A post hoc analysis of samples from men with metastatic castrate-resistant prostate cancer (mCRPC) participating in a randomized clinical trial demonstrated a benefit with the addition of carboplatin to cabazitaxel in those patients whose samples had AVPCm detected via immunohistochemistry (AVPCm^IHC^) and/or circulating tumor DNA (AVPCm^ctDNA^), though the concordance of IHC and ctDNA was low and available samples were limited [15].

*TP53* alterations in prostate cancer are often missense mutations within the DNA-binding domain, resulting in p53 nuclear accumulation in IHC [16,17,18]. Conversely, common *RB1* and *PTEN* alterations in prostate cancer are copy number losses which manifest as negative expression in IHC [19,20]. Previous studies have described IHC as a poor method for detecting p53 copy number losses or nonsense/frameshift/indel alterations, while RB1 and PTEN protein expression loss may occur despite the absence of hemizygous or homozygous allelic loss [16,19,20,21,22]. In a more recent study using IHC, RNA microarray expression profiling, and DNA-targeted next-generation sequencing (NGS) for the evaluation of AVPCm in 28 prostate cancer patient-derived xenografts, we found that NGS had only slightly higher agreement scores with the loss-of-function transcriptional scores for *TP53* compared to IHC, and both modalities had similar agreement to the loss-of-function transcriptional scores for *RB1* and *PTEN* [23].

The purpose of this study was to determine the feasibility and performance of various assays for identifying AVPCm in clinical patient samples using CLIA-certified assays to inform its use as a selection or stratification factor in prospective clinical trials. Patients with progressive mCRPC who were undergoing metastatic tumor biopsies as a standard of care were prospectively enrolled to measure the inter-reader variability, inter-assay agreement, and turnaround times for each of the AVPCm components as detected in IHC and NGS assays of solid tumor biopsies (stDNA) and NGS for plasma-derived ctDNA.

## 2. Materials and Methods

### 2.1. Study Design and Sample Collection

Men with mCRPC seen at the MD Anderson Cancer Center (MDACC) undergoing standard-of-care tumor biopsies at sites of metastasis for disease progression were eligible for enrollment. Formalin-fixed paraffin-embedded (FFPE) biopsy samples were subjected to IHC analysis and stDNA NGS for standard clinical care. Consenting patients concurrently donated two 10 mL Streck tubes (La Vista, NE, USA) via venipuncture for plasma ctDNA analyses. The date and time of each collection was recorded, and the samples were barcoded with a de-linked unique sample ID number and shipped at room temperature to the Genetics and Solid Tumor Laboratory at the University of Washington for processing and analysis. The confidentiality of patient data was maintained using the Prometheus Software Platform (version 1.0), a secure, password-protected, 21CFR part 11-compliant database that can only be accessed by investigators involved in the study. The study protocol was approved by the Institutional Review Board (IRB) and conducted in accordance with the precepts established by the Helsinki Declaration. Before enrollment, all patients signed IRB-approved written informed consent forms.

### 2.2. Immunohistochemistry: AVPCm^IHC^

The FFPE samples from the metastatic tumor biopsies were processed at the CLIA-certified MD Anderson Clinical Pathology Laboratory as per the clinical standard of care. An H&E slide was reviewed by the pathologist to determine tumor content. Immunohistochemistry was performed with antibodies for TP53 (Leica Microsystems, Deer Park, IL, USA), RB1 (Calbiochem, Burlington, MA, USA), and PTEN (Agilent Dako, Santa Clara, CA, USA). Inter-assay concordance measures were measured using the results provided on the clinical pathology report in the patient’s electronic medical record. To assess inter-reader variability, the sections stained for p53, RB1, and PTEN were reviewed by two independent pathologists (Rev1 and Rev2). The antibody identification and epitope mapping are described in Supplementary Table S1 and Supplementary Figure S1. During the study, the RB1 antibody was changed from Calbiochem/OP66 to BD/554136 (BD Biosciences, Franklin Lakes, NJ, USA) due to unavailability from the vendor. Both antibody stains used appropriate controls and followed well-standardized protocols. The labeling index (Li, percentage of positive cells) was calculated as the number of positively stained epithelial cells divided by the total number of epithelial cells, at X200 magnification. The percentage of malignant cells with nuclear (RB1 and TP53) or cytoplasmic (PTEN) staining was recorded using the following intensity levels: 0 (no staining), 1+ (weak staining), 2+ (moderate staining), and 3+ (strong staining), as previously described [12]. Based on previous findings, the Li for Tp53 and RB1 was summed over 2+ and 3+, while PTEN was summed over 1+, 2+, and 3+. RB1 and PTEN were considered aberrant if the Li ≤ 10%, and Tp53 was considered aberrant if Li ≥ 10%, as previously described [12,23]. Samples were considered AVPCm^IHC+^ if aberrant results were observed for at least 2 of Tp53, RB1, and PTEN.

### 2.3. Next-Generation Sequencing of FFPE Tumor DNA: AVPCm^stDNA^

The next-generation sequencing (NGS) of stDNA was performed at MD Anderson’s CLIA-certified molecular diagnostics laboratory, to screen for alterations in *TP53*, *RB1*, and *PTEN* as part of the routine clinical work-up. During the course of this study, MD Anderson’s NGS platform was switched from the STGA (Solid Tumor Genomic Assay) to the MDA-MAPP™ (MD Anderson Mutation Analysis Precision Panel™) assay, both custom in-house developed assays. The switch facilitated (i) a larger, more comprehensive assay that includes >600 genes, microsatellite instability status (MSI), and tumor mutational burden (TMB) information, and (ii) faster turn-around times by reducing dependence on outside vendors.

For the STGA, the genomic reference sequence used was GRCh37/hg19. The assay was designed to detect point mutations, small insertions/deletions, and copy number gains (amplifications). Matched non-tumor tissues from patients were tested and germline variants were excluded. Variants detected at very low allelic frequencies that were not deemed to be confirmable by independent, orthogonal methods and/or in significant discordance with the percentage of tumor in the tested sample were excluded. For clinical purposes, the effective lower limit of detection of this assay (analytical sensitivity) for single-nucleotide variations was determined to be in the range of 5% (one mutant allele in the background of nineteen wild-type alleles) to 10% (one mutant allele in the background of nine wild-type alleles) by taking into consideration the depth of coverage at a given base and the ability to confirm low-level mutations using independent conventional platforms. A minimum of 20% tumor nuclei in the sample was required to reduce the potential for false-negative results. The analytic pipeline in this assay attempts to normalize for inter-amplicon performance differences and total sample loading, but does not attempt to correct for tumor percentage.

The MDA-MAPP™ assay uses targeted hybridization-based capture technology for the detection of sequence variants/mutations in 610 genes, amplifications in 583 genes, select gene rearrangements in 34 genes, and select genomic immuno-oncology signatures including MSI and TMB in DNA isolated from FFPE tumor tissue and cytology specimens. MDA-MAPP™ employs DNA extracted from both tumor tissue and paired normal (blood or tissue) specimens in the CLIA-certified molecular diagnostic laboratory. A minimum of 50 ng of genomic DNA undergoes whole-genome library construction with adapters carrying unique molecular indices, allowing for the tagging of original double-stranded DNA that facilitates the statistical reconstruction of reads sequenced as duplicates from a single-amplified genome. A target area of 2.1 megabases of the hg19 genome is enriched with custom, hybrid-capture, 120nt dsDNA probes. MDA-MAPP™ uses the NovaSeq 6000 NGS (Illumina, San Diego, CA, USA) platform and bidirectional paired-end sequencing to identify nucleic acid variants for all coding regions from most genes in the panel, the *TERT* promoter, 1 non-coding RNA gene, and clinically relevant rearrangements. Reported somatic mutations are identified through a comparison to the human genome reference sequence, GRCh37/hg19, and reviewed in OncoSeek against a process-matched normal control. Data analysis is performed in-house using the MDA-MAPP™ Bioinformatics pipeline, which relies on the dual-duplex molecular barcoding for consensus analysis to reduce sequencing artifacts and achieve greater sensitivity and positive predictive value.

Of note, copy number losses for MDA-MAPP™ were not validated as part of the CLIA assay, so no threshold for deletions had been defined. We retrospectively interpreted deletion CNVs using a copy number threshold ≤1.5 based on sequencing normal FFPE samples and exploring levels of noise in copy number estimates.

### 2.4. Next-Generation Sequencing of Circulating Tumor DNA: AVPCm^ctDNA^

At the University of Washington Genetics and Solid Tumor Laboratory, germline DNA was extracted from 1 mL of each sample and ctDNA was extracted from the remaining 9 mL for testing using the UW-OncoPlex (University of Washington, Seattle, WA, USA) to provide simultaneous deep-sequencing information, based on a >500× average coverage, for all classes of mutations in 194 clinically relevant genes [24,25]. Genomic alterations (defined as for AVPCm^stDNA^) in ≥ 2 of *TP53*, *RB1*, and/or *PTEN* were considered AVPCm^ctDNA+^. ctDNA fractions estimated at less than 1% or that had no detected somatic mutations were considered indeterminate and could represent false negatives.

### 2.5. Outcome Variables and Statistical Analysis

Fifty-four patients were targeted in order to have 80% power to detect a Cohen’s Kappa of 0.8 as significantly greater than 0.5 at a two-sided significance level of 0.05 [26]. We expected approximately one third of patients to have AVPCm^+^ tumors. Concordance between the two pathologists was estimated based on Cohen’s Kappa and a 95% confidence interval for each molecular marker as well as the AVPCm^IHC^. Cohen’s Kappa and 95% confidence intervals were also used to determine the inter-assay agreement between each of the three assays: IHC, stDNA, and ctDNA. The turnaround times for each of the assays were evaluated using descriptive statistics.

## 3. Results

### 3.1. Sample Evaluability

**Figure 1:**
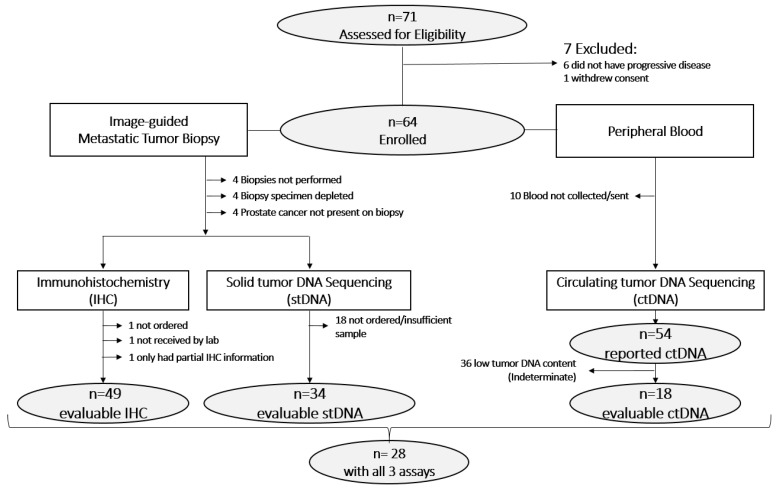
Consort diagram.

Due to a suboptimal number of evaluable samples, the protocol was amended to enroll 10 additional patients to a total of 64. We reviewed 71 patients between August 2020 and March 2022 (Figure 1). Six patients were ineligible due to specimens obtained in the absence of disease progression and one patient rescinded consent. Of the 64 eligible patients, results were obtained for all three tumor suppressors in 49 (77%) via IHC, 34 (53%) via stDNA NGS, and 54 (84%) via ctDNA NGS. Table 1 lists the biopsy sites and sample evaluability for each of the assays.

### 3.2. Immunohistochemistry Results and Inter-Reader Variability

**Figure 2:**
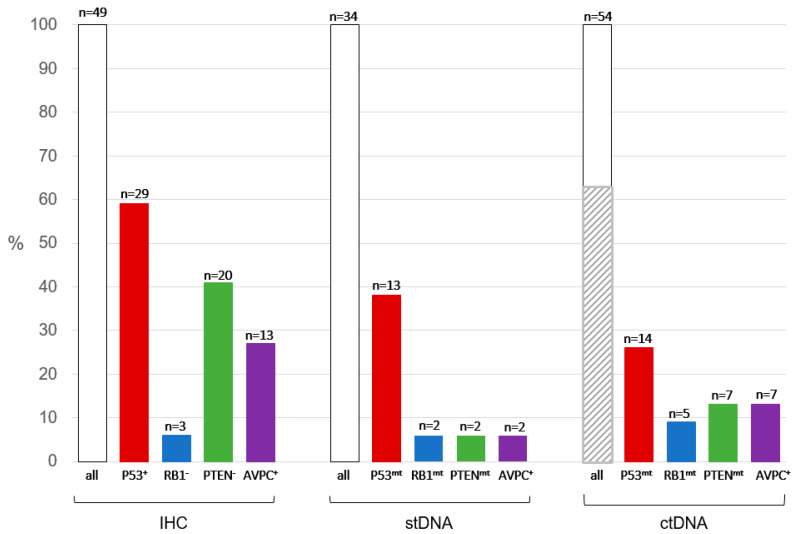
Tp53, RB1, and PTEN mutation rates and AVPCm positivity by assay: IHC, stDNA, and ctDNA. Note that CNV results for stDNA are not included. Shaded region is samples with insufficient ctDNA tumor content to avoid risk of false negatives.

Absent IHC results were due to urothelial cancer on pathology (*n* = 1), benign tissue on pathology (*n* = 3), tissue block depleted (*n* = 2), insufficient tumor on pathology (*n* = 4), tissue not requested/received (*n* = 2), and biopsy not performed (*n* = 3). As mentioned in the Methods section, the RB1 antibody was changed during our study. Three (20%) and four (27%) of 15 samples stained with the Calbiochem/OP66 antibody and six (18%) and eight (24%) of 34 samples stained with the BD/554136 antibody were read as RB1-aberrant by Rev1 and Rev 2, respectively. Of the 49 samples evaluable for IHC, 13 (27%) met the AVPCm^IHC^ criteria (see Figure 2 and Table S2).

There was disagreement in the Tp53 aberrancy calls for six (12.2%) samples, such that it was close to the target of 0.8 with lower bounds of the CI above 0.5 (Kappa = 0.76 (95% CI: 0.58, 0.94)) (Table 2). There was lower agreement for the RB1 results (Kappa = 0.58 (95% CI: 0.31,0.86)) with disagreement for seven (14.3%) samples overall. In contrast, the PTEN inter-reader agreement was high (Kappa = 0.91 (95% CI: 0.79, 1.00)) with disagreement for only two (4.1%) samples. In terms of AVPCm^IHC^ detection, the CI lower bound fell below the desired threshold of 0.50 (Kappa = 0.66 (95% CI: 0.43, 0.89)), with disagreement for seven (14.3%) samples (Table 2).

### 3.3. Next-Generation Sequencing of stDNA

NGS of stDNA was absent in 17 samples due to inadequate tissue or request failure. As mentioned in the Methods section, the NGS platform was switched during the course of our study. Of the 21 samples analyzed with the MDA-146 assay, genomic alterations were reported in six (29%) for Tp53, one (5%) for RB1, and two (6%) for PTEN. Of the 13 samples analyzed with the MAPP assay, genomic alterations were reported in seven (54%) for Tp53, one (8%) for RB1, and zero (0%) for PTEN. As noted previously, CNV detection was not validated as part of the MAPP assay. With the retrospective CNV deletion analysis (described in Methods), CNVs and SNVs were observed in 11 (32%) and 13 (38%) samples for TP53, 16 (47%) and 2 (6%) for RB1, and 16 (47%) and 2 (6%) for PTEN, respectively. Of the 34 samples evaluable for stDNA, two (6%) met the AVPCm^stDNA^ criteria. When the retrospective CNV analysis was included, this number increased to 15 (44%) samples.

### 3.4. Next-Generation Sequencing of ctDNA

For the ctDNA analysis, 10 samples were not collected/sent due to a lack of coordination with the tissue biopsies. Fourteen (25.9%) patients had detectable CNV (*n* = 2), SNV (*n* = 11), or both (*n* = 1) in TP53. Five (9.2%) patients had detectable CNV (*n* = 4) or SNV (*n* = 1) in RB1. Seven (13.0%) patients had detectable CNV (*n* = 6) or SNV (*n* = 1) in PTEN. Notably, 35 (64.8%) of the 54 ctDNA samples were reported to have low tumor content (i.e., indeterminate samples), such that deleterious mutations may not have been detected (i.e., false negatives could not be confidently ruled out). However, three indeterminate ctDNA samples did detect mutations, all of which were TP53 SNVs. Overall, the AVPCm^ctDNA^ was detected in seven (13.0%) of the 54 samples.

### 3.5. Inter-Assay Agreements

Table 3 presents the frequencies and concordance for AVPCm^+^ calls by the various assays. IHC and ctDNA had agreement in 33 (78.6%) of 42 patients, with four (9.5%) that were called AVPCm^+^ for both assays (Kappa = 0.35 (95% CI: 0.03, 0.67)). There was significant discordance when measuring the agreement between stDNA and IHC (Kappa 0.07 (95% CI: −0.19, 0.33)) as well as stDNA and ctDNA (Kappa 0.20 (95% CI: −0.24, 0.65)) (Table 3A). When non-CLIA copy number loss analyses for TP53, RB1, and PTEN were included, the concordance between AVPCm^IHC^ and AVPCm^stDNA adjusted^ improved with Kappa 0.30 (95% CI: −0.02, 0.61) and Kappa 0.32 (95% CI: 0.07, 0.57), respectively (Table 3A). When limiting to ctDNA samples that were deemed sufficient for analysis by excluding those with a low tumor content, the concordance improved between AVPCm^ctDNA^ and AVPCm^IHC^ (Kappa 0.57 (95% CI: 0.15, 1.00)) as well as between AVPCm^ctDNA^ and AVPCm^stDNA adjusted^ (Kappa 0.65 (95% CI: 0.23, 1.00)) (Table 3B). See Table S3 for details regarding the inter-assay agreement for each tumor suppressor.

### 3.6. Assay Turnaround Times

Turnaround times were measured from the date the order was placed to the date the report was released. Median (and range) turnaround time was 12 days (2–35) for IHC, 28 days (12–56) for stDNA and 16 days (11–56) for ctDNA. (Supplementary Figure S2) Median (and range) time to biopsy from date of registration was 4 days (1–42).

## 4. Discussion

In this prospective study, we show the operational characteristics of AVPCm detection using CLIA-certified FFPE IHC, FFPE stDNA NGS, and ctDNA NGS assays in routine clinical practice. As future studies assess the efficacy of novel therapeutics in mCRPC, these data inform the design of trials that use AVPCm as a criterion for patient selection or stratification.

Only 28 (43%) enrolled patients had evaluable samples with all three assays despite restricting enrollment to patients with progressive metastatic disease. ctDNA had the highest proportion of reported results (84%), though only 58% of patients had a sufficient tumor content. Liver biopsies had a higher percentage of sufficient ctDNA tumor content as opposed to bone biopsies, suggesting an enrichment of more aggressive disease that is better detected in a sufficient amount in the circulation and aligns with prior publications [27]. IHC had the next highest proportion of evaluable samples (77%), though a sizable portion could not be evaluated due to the depletion of FFPE blocks or the absence of cancer in the sample. This was likely explained in part due to the large percentage of bone biopsies (30%); 57% of depleted FFPE blocks or samples with absent cancer in the biopsy sample were from bone biopsies. In addition, stDNA had logistical issues leading to a further suboptimal yield, as many samples did not undergo sequencing because the treating physician failed to place the order for molecular profiling at the time the biopsy was obtained, and patients later deteriorated or did not return for care, such that orders could not be placed. stDNA also had the longest turnaround time (median 28 days) compared to IHC or ctDNA.

In terms of utility, AVPCm was detected in 13 (27%) patients by IHC, two (6%) by stDNA NGS, andseven7 (13%) by ctDNA NGS. Herberts et al. performed NGS on plasma and synchronous metastatic tissue samples collected from patients with mCRPC. They describe a median ctDNA fraction of 47% (17–82%) and detected a significant number of inactivating somatic alterations in *TP53* (73%), *RB1* (18%), and *PTEN* (48%) [28]. It should be noted that these samples were selected retrospectively, which may explain the overall high yield. In our prospective study, ctDNA detected the greatest proportion of AVPCm^+^ tumors (38.9%) but were indeterminate in 66.7% of samples, despite being obtained in a cohort of patients with progressing metastatic disease. IHC had the second highest detection rate of AVPCm^+^ tumors along with a reasonable 12-day median turnaround time, with a wide range observed due more to delays in carrying out the order than to the actual time needed to perform the assay. Though feasible in terms of sample collection and turnaround time, concordance of IHC reads between pathologists was variable: Kappa 0.76, 0.58, and 0.91 for Tp53, RB1, and PTEN, respectively. A potential limitation is our selection of Li cutoffs and intensity measures based on prior experience [12]; other cutoffs may yield better concordance. RB1 was particularly discordant, which may have contributed to the AVPCm concordance that was less than the CI lower bound desired threshold. IHC and ctDNA had agreement in 33/42 patients, with four who were AVPCm^+^ for both measures (Kappa = 0.35 (95% CI: 0.03, 0.67)). This measure was, to some extent, affected by the high number of ctDNA samples with indeterminate results due to a low tumor content, as the Kappa increased from 0.35 to 0.57 when only assessing samples with sufficient ctDNA. This is notable, as ctDNA assays, even those that are well validated for the ultra-low detection of SNVs, have difficulties in accurately detecting CNVs in the setting of a low tumor content, which is the dominant molecular mechanism to diagnose AVPCm.

stDNA had agreement with neither IHC nor ctDNA (Kappa 0.07 and 0.20, respectively). The Kappa only modestly improved when excluding indeterminate ctDNA samples due to a low tumor content. This is likely due to copy number loss not being reported for the MAPP stDNA assay. When we included a non-CLIA analysis of deletion CNVs (stDNA-adjusted), the Kappa improved with IHC and ctDNA. However, RB1 aberrancy calls had less agreement between stDNA-adjusted and IHC/ctDNA compared to stDNA, while PTEN improved and P53 remained similar. Commonly used commercial NGS platforms such as FoundationOne^®^CDx and Tempus xT are also performed on FFPE samples and reportedly detect, along with SNVs, insertions/deletions and copy number alterations for *TP53*, *RB1*, and *PTEN,* which may have demonstrated better tumor suppressor aberrancy and AVPCm detection [29,30]. Our findings suggests that FFPE tumor NGS may serve as the optimal assay if it is well-validated to detect copy number losses and can accurately distinguish mono- vs. bi-allelic loss.

An important finding is the low inter-assay concordance of IHC and DNA sequencing detected when taken together with the findings in our previous studies of PDX models, which also showed a gap between IHC and DNA results. This suggests that the biological and clinical meaning of each method is likely different. For example, genomic alterations in *RB1* and *PTEN* may simply be a reflection of an overall high burden of copy number alterations, while IHC may be more reflective of a distinct biology. One can explain a genomically normal marker and an aberrant IHC by considering transcriptional and posttranslational modifications. Tumor heterogeneity and extrachromosomal DNA may explain normal IHC despite a copy number loss. This may be better answered by integrated single cell sequencing studies and spatial proteomic analyses.

The strengths of our study are its prospective measure of AVPCm using CLIA-certified laboratory assays in real-world patients with progressive mCRPC receiving routine care, as well as the measure of inter-reader IHC agreement and inter-assay agreement for predefined aberrancies in markers of interest. Importantly, the significant proportion of ctDNA samples with a low tumor content informs the utility of ctDNA use for the purposes of clinical trial selection or stratification. A weakness of our study is the single-institution design, which limits the external validity of the observed causes for the suboptimal number of evaluable samples and the measured turnaround times. Another limitation is the use of institutional stDNA NGS platforms with limited CNV detection, which did not account for more commonly used commercial platforms. Lastly, the change in the RB1 antibody presents a potential weakness, though this change was reflective of real-world occurrences in clinical practice.

## 5. Conclusions

In sum, we show the operational characteristics of detecting the AVPCm using CLIA-certified assays in routine clinical practice. IHC had a higher detection rate of AVPCm^+^ tumors and the shortest turnaround times, making it an attractive choice for use as a selection or stratification tool in prospective clinical trials. However, the high inter-reader variability in its assessment calls for the inclusion of a central review of results in clinical trial designs. The challenges of copy number loss detection in stDNA and low tumor content in ctDNA may well be overcome by technological advances. These data inform the design of clinical trials testing the ability of AVPCm^IHC^, AVPCm^DNA^, or AVPCm^IHC/DNA^ to contribute to a much-needed, clinically meaningful, therapeutically relevant, and biologically based classification of prostate cancer.

## Figures and Tables

**Table 1:** Biopsy characteristics by availability for each AVPC measure.

		All Patients		IHC Evaluable *		stDNA Evaluable		ctDNA Evaluable		ctDNA Sufficient **	
Patient Characteristics		N	(%)	N	(%)	N	(%)	N	(%)	N	(%)
All		64	(100%)	49	(100%)	34	(100%)	54	(100%)	19	(100%)
Age—median (min, max)		66.5	(48, 86)	66	(48, 86)	66	(48, 86)	67	(48, 86)	65	(48, 75)
Biopsy Year	2020	12	(19%)	11	(22%)	10	(29%)	10	(19%)	5	(26%)
	2021	47	(73%)	36	(73%)	23	(68%)	41	(76%)	12	(63%)
	2022	2	(3%)	2	(4%)	1	(3%)	2	(4%)	1	(5%)
	None	3	(5%)	0	(0%)	0	(0%)	1	(2%)	1	(5%)
Biopsy Site	Not Applicable	3	(5%)	0	(0%)	0	(0%)	1	(2%)	1	(5%)
	Adrenal	1	(2%)	1	(2%)	0	(0%)	1	(2%)	0	(0%)
	Bladder	1	(2%)	0	(0%)	0	(0%)	1	(2%)	0	(0%)
	Bone	19	(30%)	15	(31%)	10	(29%)	15	(28%)	2	(11%)
	Bone marrow	1	(2%)	0	(0%)	0	(0%)	1	(2%)	0	(0%)
	LN	23	(36%)	21	(43%)	15	(44%)	20	(37%)	6	(32%)
	Liver	11	(17%)	8	(16%)	8	(24%)	11	(20%)	9	(47%)
	Lung	3	(5%)	2	(4%)	0	(0%)	3	(6%)	1	(5%)
	Prostate	2	(3%)	2	(4%)	1	(3%)	1	(2%)	0	(0%)
IHC Evaluable	No	15	(23%)	0	(0%)	2	(6%)	12	(22%)	4	(21%)
	Yes	49	(77%)	49	(100%)	32	(94%)	42	(78%)	15	(79%)
stDNA Evaluable	No	30	(47%)	17	(35%)	0	(0%)	26	(48%)	8	(42%)
	Yes	34	(53%)	32	(65%)	34	(100%)	28	(52%)	11	(58%)
ctDNA Evaluable	No	10	(16%)	7	(14%)	6	(18%)	0	(0%)	0	(0%)
	Yes	54	(84%)	42	(86%)	28	(82%)	54	(100%)	19	(100%)

* A total of 50 patients had any IHC, but one patient had partial IHC information that did not allow assessment of AVPC, so only 49 are counted as available. ** Excludes ctDNA samples that had low tumor content resulting in indeterminate findings that cannot rule out false negatives.

**Table 2:** Concordance for Tp53 (2+/3+), RB (2+/3+), and PTEN abnormalities and for AVPC status. IHC: immunohistochemistry; Rev1: pathologist 1; Rev2: pathologist 2.

Tp53 Abnormal		Rev2, *n*		Kappa (95% CI)
		No	Yes	
Rev1, *n*	No	21	5	0.76 (0.58, 0.94)
	Yes	1	23	
RB1 abnormal		Rev2, *n*		
		No	Yes	
Rev1, *n*	No	36	2	0.58 (0.31, 0.86)
	Yes	5	7	
PTEN abnormal		Rev2, *n*		
		No	Yes	
Rev1, *n*	No	31	1	0.91 (0.79, 1.00)
	Yes	1	16	
AVPCm^IHC^		Rev2, *n*		
		No	Yes	
Rev1, *n*	No	32	4	0.66 (0.43, 0.89)
	Yes	3	11	

**Table 3:** (**A**) Concordance between assays for AVPCm aberrancy calls. (**B**) Concordance between assays for AVPCm aberrancy calls (sufficient ctDNA samples only).

A				
AVPCm^IHC^ vs. AVPCm^ctDNA^				
	AVPCm^ctDNA−^	AVPCm^ctDNA+^	total	Kappa (95% CI)
AVPCm^IHC−^	29	2	31	0.35 (0.03, 0.67)
AVPCm^IHC+^	7	4	11	
total	36	6	42	
AVPCm^IHC^ vs. AVPCm^stDNA^				
	AVPCm^stDNA−^	AVPCm^stDNA+^	total	Kappa (95% CI)
AVPCm^IHC−^	21	1	22	0.07 (−0.19, 0.33)
AVPCm^IHC+^	9	1	10	
total	30	2	32	
AVPCm^stDNA^ vs. AVPCm^ctDNA^				
	AVPCm^ctDNA−^	AVPCm^ctDNA+^	total	Kappa (95% CI)
AVPCm^stDNA−^	22	4	26	0.20 (−0.24, 0.65)
AVPCm^stDNA+^	1	1	2	
total	23	5	28	
AVPCm^stDNA adjusted^ vs. AVPCm^IHC^				
	AVPC^stDNA adjusted−^	AVPC^stDNA adjusted+^	total	Kappa (95% CI)
AVPC^IHC−^	14	8	22	0.30 (−0.02, 0.61)
AVPC^IHC+^	3	7	10	
Total	17	15	32	
AVPCm^stDNA adjusted^ vs. AVPCm^ctDNA^				
	AVPCm^ctDNA−^	AVPCm^ctDNA+^	total	Kappa (95% CI)
AVPC^stDNA adjusted−^	13	0	13	0.32 (0.07, 0.57)
AVPC^stDNA adjusted+^	10	5	15	
total	23	5	28	
**B**				
**AVPCm^IHC^ vs. AVPCm^ctDNA^**				
	AVPCm^ctDNA−^	AVPCm^ctDNA+^	total	Kappa (95% CI)
AVPCm^IHC−^	8	2	10	0.57 (0.15, 1.00)
AVPCm^IHC+^	1	4	5	
total	9	6	15	
AVPCm^IHC^ vs. AVPCm^stDNA^				
	AVPCm^stDNA−^	AVPCm^stDNA+^	total	Kappa (95% CI)
AVPCm^IHC−^	6	0	6	0.21 (−0.17, 0.59)
AVPCm^IHC+^	4	1	5	
total	10	1	11	
AVPCm^stDNA^ vs. AVPCm^ctDNA^				
	AVPCm^ctDNA−^	AVPCm^ctDNA+^	total	Kappa (95% CI)
AVPCm^stDNA−^	6	4	10	0.21 (−0.17, 0.59)
AVPCm^stDNA+^	0	1	1	
total	6	5	11	
AVPCm^IHC^ vs. AVPCm^stDNA adjusted^				
	AVPC^stDNA adjusted−^	AVPC^stDNA adjusted+^	total	Kappa (95% CI)
AVPC^IHC−^	3	3	6	0.29 (−0.23, 0.81)
AVPC^IHC+^	1	4	5	
total	4	7	11	
AVPCm^stDNA adjusted^ vs. AVPCm^ctDNA^				
	AVPCm^ctDNA−^	AVPCm^ctDNA+^	total	Kappa (95% CI)
AVPC^stDNA adjusted−^	4	0	4	0.65 (−0.23, 1.00)
AVPC^stDNA adjusted+^	2	5	7	
total	6	5	11	

## Data Availability

The data presented in this study are available on request from the corresponding author. The data are not publicly available due to privacy concerns.

## Supplementary Materials

The following supporting information can be downloaded at https://www.mdpi.com/article/10.3390/cancers15245843/s1: Figure S1: Epitopes recognized by various antibodies used for immunohistochemical staining of the three tumor suppressor proteins (TSPs), TP53, RB1 and PTEN.; Table S1: Details of antibodies used for immunohistochemical staining of TP53, RB1 and PTEN, along with the antigen-retrieval protocol used by each of the three laboratories employed in this study in the CLIA-certified clinical laboratory at MD Anderson Cancer Center (MDACC). Table S2: Association between two pathologists for labeling index of Tp53 (2+/3+), RB (2+/3+), and PTEN (1+/2+/3+). Table S3A: Concordance between assays for tumor suppressor aberrancy calls. Table S3B: Concordance between assays for tumor suppressor aberrancy calls (sufficient samples only). Figure S2: Turnaround Times.
