# Supplementary material for: Evaluation of the Aggressive-Variant Prostate Cancer Molecular Signature in Clinical Laboratory Improvement Amendments (CLIA) Environments
Source: Cancers (Basel). 2023 Dec 14;15(24):5843. doi: 10.3390/cancers15245843 (PMC10741546; doi:10.3390/cancers15245843)
Supplement: Supplementary file 1 [file cancers-15-05843-s001.zip › Supplementary Table S1.pptx]

## Slide 1
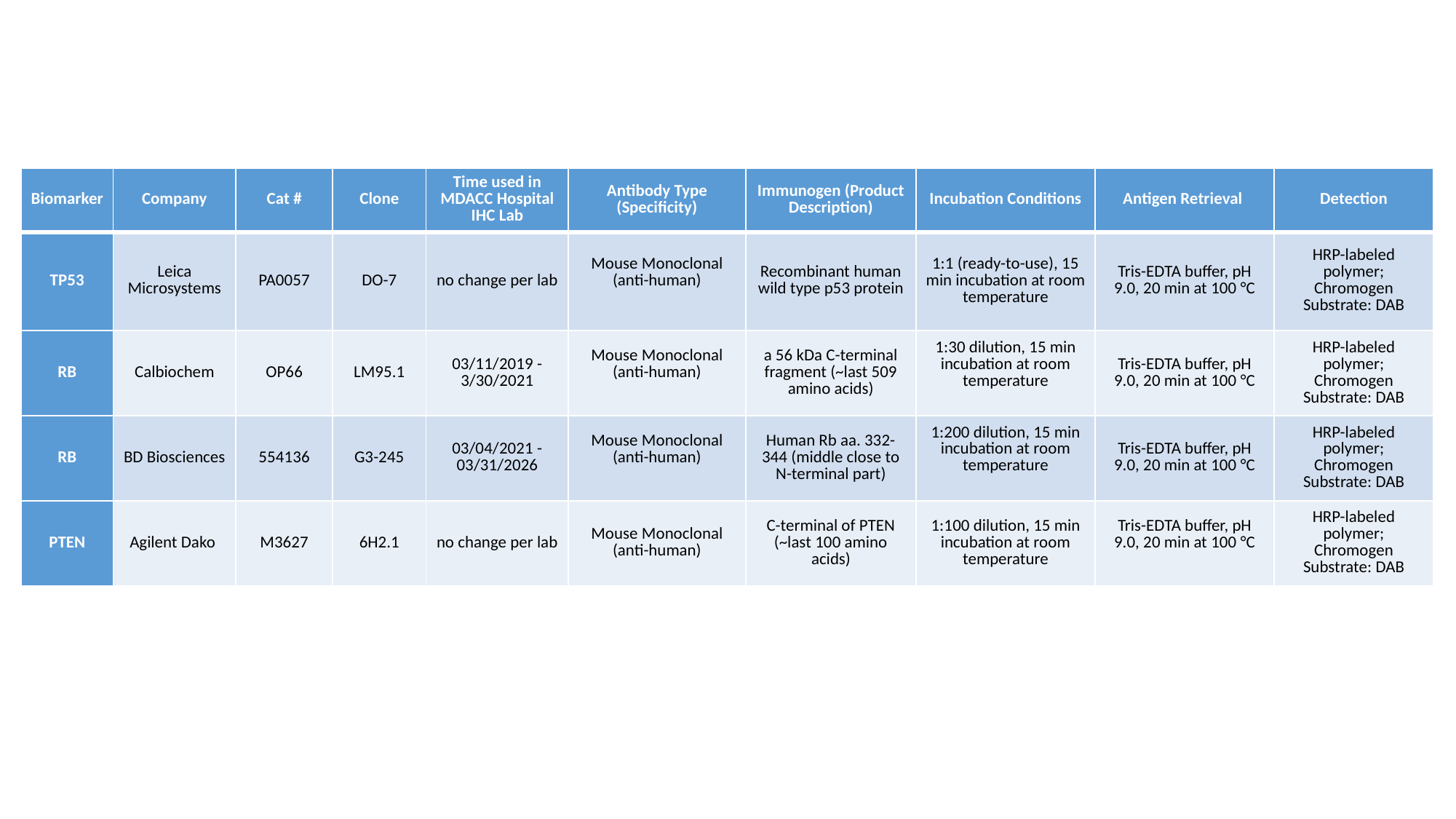

| Biomarker | Company | Cat # | Clone | Time used in MDACC Hospital IHC Lab | Antibody Type (Specificity) | Immunogen (Product Description) | Incubation Conditions | Antigen Retrieval | Detection |
| --- | --- | --- | --- | --- | --- | --- | --- | --- | --- |
| TP53 | Leica Microsystems | PA0057 | DO-7 | no change per lab | Mouse Monoclonal (anti-human) | Recombinant human wild type p53 protein | 1:1 (ready-to-use), 15 min incubation at room temperature | Tris-EDTA buffer, pH 9.0, 20 min at 100 °C | HRP-labeled polymer; Chromogen Substrate: DAB |
| RB | Calbiochem | OP66 | LM95.1 | 03/11/2019 - 3/30/2021 | Mouse Monoclonal (anti-human) | a 56 kDa C-terminal fragment (~last 509 amino acids) | 1:30 dilution, 15 min incubation at room temperature | Tris-EDTA buffer, pH 9.0, 20 min at 100 °C | HRP-labeled polymer; Chromogen Substrate: DAB |
| RB | BD Biosciences | 554136 | G3-245 | 03/04/2021 - 03/31/2026 | Mouse Monoclonal (anti-human) | Human Rb aa. 332-344 (middle close to N-terminal part) | 1:200 dilution, 15 min incubation at room temperature | Tris-EDTA buffer, pH 9.0, 20 min at 100 °C | HRP-labeled polymer; Chromogen Substrate: DAB |
| PTEN | Agilent Dako | M3627 | 6H2.1 | no change per lab | Mouse Monoclonal (anti-human) | C-terminal of PTEN (~last 100 amino acids) | 1:100 dilution, 15 min incubation at room temperature | Tris-EDTA buffer, pH 9.0, 20 min at 100 °C | HRP-labeled polymer; Chromogen Substrate: DAB |
