# Supplementary material for: Evaluation of the Aggressive-Variant Prostate Cancer Molecular Signature in Clinical Laboratory Improvement Amendments (CLIA) Environments
Source: Cancers (Basel). 2023 Dec 14;15(24):5843. doi: 10.3390/cancers15245843 (PMC10741546; doi:10.3390/cancers15245843)
Supplement: Supplementary file 1 [file cancers-15-05843-s001.zip › Supplementary Table S2.pptx]

## Slide 1
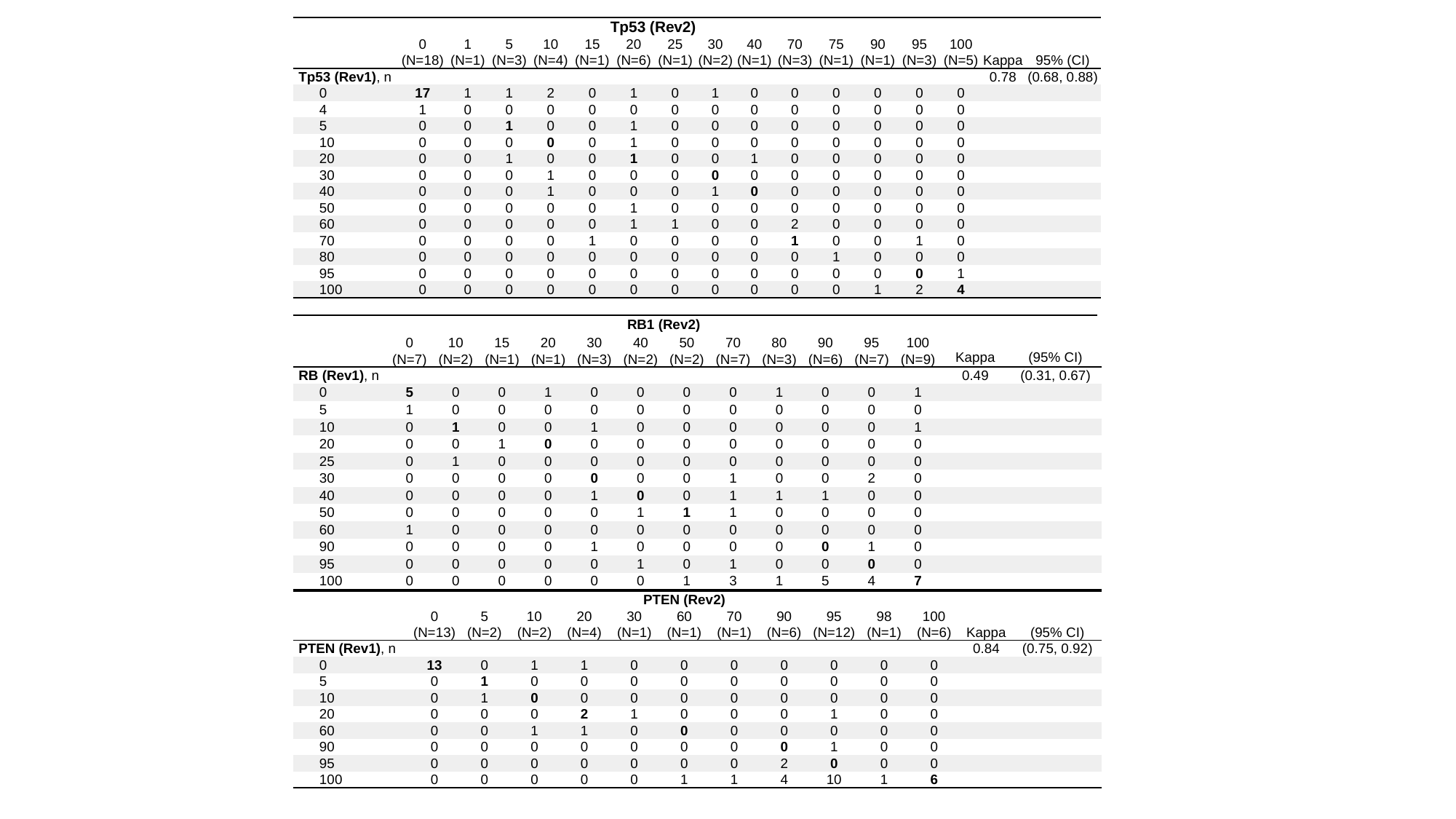

| Tp53 (Rev2) | | | | | | | | | | | | | | | | | | |
| --- | --- | --- | --- | --- | --- | --- | --- | --- | --- | --- | --- | --- | --- | --- | --- | --- | --- | --- |
| | 0 (N=18) | 1 (N=1) | 5 (N=3) | 10 (N=4) | 15 (N=1) | 20 (N=6) | 25 (N=1) | 30 (N=2) | | 40 (N=1) | | 70 (N=3) | 75 (N=1) | 90 (N=1) | 95 (N=3) | 100 (N=5) | Kappa | 95% (CI) |
| Tp53 (Rev1), n | | | | | | | | | | | | | | | | | 0.78 | (0.68, 0.88) |
| 0 | 17 | 1 | 1 | 2 | 0 | 1 | 0 | 1 | | 0 | | 0 | 0 | 0 | 0 | 0 | | |
| 4 | 1 | 0 | 0 | 0 | 0 | 0 | 0 | 0 | | 0 | | 0 | 0 | 0 | 0 | 0 | | |
| 5 | 0 | 0 | 1 | 0 | 0 | 1 | 0 | 0 | | 0 | | 0 | 0 | 0 | 0 | 0 | | |
| 10 | 0 | 0 | 0 | 0 | 0 | 1 | 0 | 0 | | 0 | | 0 | 0 | 0 | 0 | 0 | | |
| 20 | 0 | 0 | 1 | 0 | 0 | 1 | 0 | 0 | | 1 | | 0 | 0 | 0 | 0 | 0 | | |
| 30 | 0 | 0 | 0 | 1 | 0 | 0 | 0 | 0 | | 0 | | 0 | 0 | 0 | 0 | 0 | | |
| 40 | 0 | 0 | 0 | 1 | 0 | 0 | 0 | 1 | | 0 | | 0 | 0 | 0 | 0 | 0 | | |
| 50 | 0 | 0 | 0 | 0 | 0 | 1 | 0 | 0 | | 0 | | 0 | 0 | 0 | 0 | 0 | | |
| 60 | 0 | 0 | 0 | 0 | 0 | 1 | 1 | 0 | | 0 | | 2 | 0 | 0 | 0 | 0 | | |
| 70 | 0 | 0 | 0 | 0 | 1 | 0 | 0 | 0 | | 0 | | 1 | 0 | 0 | 1 | 0 | | |
| 80 | 0 | 0 | 0 | 0 | 0 | 0 | 0 | 0 | | 0 | | 0 | 1 | 0 | 0 | 0 | | |
| 95 | 0 | 0 | 0 | 0 | 0 | 0 | 0 | 0 | | 0 | | 0 | 0 | 0 | 0 | 1 | | |
| 100 | 0 | 0 | 0 | 0 | 0 | 0 | 0 | 0 | | 0 | | 0 | 0 | 1 | 2 | 4 | | |
| | RB1 (Rev2) | | | | | | | | | | | | | | |
| --- | --- | --- | --- | --- | --- | --- | --- | --- | --- | --- | --- | --- | --- | --- | --- |
| | 0 (N=7) | 10 (N=2) | 15 (N=1) | 20 (N=1) | 30 (N=3) | 40 (N=2) | 50 (N=2) | 70 (N=7) | 80 (N=3) | 90 (N=6) | 95 (N=7) | 100 (N=9) | Kappa | (95% CI) | |
| RB (Rev1), n | | | | | | | | | | | | | 0.49 | (0.31, 0.67) | |
| 0 | 5 | 0 | 0 | 1 | 0 | 0 | 0 | 0 | 1 | 0 | 0 | 1 | | | |
| 5 | 1 | 0 | 0 | 0 | 0 | 0 | 0 | 0 | 0 | 0 | 0 | 0 | | | |
| 10 | 0 | 1 | 0 | 0 | 1 | 0 | 0 | 0 | 0 | 0 | 0 | 1 | | | |
| 20 | 0 | 0 | 1 | 0 | 0 | 0 | 0 | 0 | 0 | 0 | 0 | 0 | | | |
| 25 | 0 | 1 | 0 | 0 | 0 | 0 | 0 | 0 | 0 | 0 | 0 | 0 | | | |
| 30 | 0 | 0 | 0 | 0 | 0 | 0 | 0 | 1 | 0 | 0 | 2 | 0 | | | |
| 40 | 0 | 0 | 0 | 0 | 1 | 0 | 0 | 1 | 1 | 1 | 0 | 0 | | | |
| 50 | 0 | 0 | 0 | 0 | 0 | 1 | 1 | 1 | 0 | 0 | 0 | 0 | | | |
| 60 | 1 | 0 | 0 | 0 | 0 | 0 | 0 | 0 | 0 | 0 | 0 | 0 | | | |
| 90 | 0 | 0 | 0 | 0 | 1 | 0 | 0 | 0 | 0 | 0 | 1 | 0 | | | |
| 95 | 0 | 0 | 0 | 0 | 0 | 1 | 0 | 1 | 0 | 0 | 0 | 0 | | | |
| 100 | 0 | 0 | 0 | 0 | 0 | 0 | 1 | 3 | 1 | 5 | 4 | 7 | | | |
| | | | | | | | | | | | | | | | |
| | PTEN (Rev2) | | | | | | | | | | | | |
| --- | --- | --- | --- | --- | --- | --- | --- | --- | --- | --- | --- | --- | --- |
| | 0 (N=13) | 5 (N=2) | 10 (N=2) | 20 (N=4) | 30 (N=1) | 60 (N=1) | 70 (N=1) | 90 (N=6) | 95 (N=12) | 98 (N=1) | 100 (N=6) | Kappa | (95% CI) |
| PTEN (Rev1), n | | | | | | | | | | | | 0.84 | (0.75, 0.92) |
| 0 | 13 | 0 | 1 | 1 | 0 | 0 | 0 | 0 | 0 | 0 | 0 | | |
| 5 | 0 | 1 | 0 | 0 | 0 | 0 | 0 | 0 | 0 | 0 | 0 | | |
| 10 | 0 | 1 | 0 | 0 | 0 | 0 | 0 | 0 | 0 | 0 | 0 | | |
| 20 | 0 | 0 | 0 | 2 | 1 | 0 | 0 | 0 | 1 | 0 | 0 | | |
| 60 | 0 | 0 | 1 | 1 | 0 | 0 | 0 | 0 | 0 | 0 | 0 | | |
| 90 | 0 | 0 | 0 | 0 | 0 | 0 | 0 | 0 | 1 | 0 | 0 | | |
| 95 | 0 | 0 | 0 | 0 | 0 | 0 | 0 | 2 | 0 | 0 | 0 | | |
| 100 | 0 | 0 | 0 | 0 | 0 | 1 | 1 | 4 | 10 | 1 | 6 | | |
| | | | | | | | | | | | | | |
