# Supplementary material for: Evaluation of the Aggressive-Variant Prostate Cancer Molecular Signature in Clinical Laboratory Improvement Amendments (CLIA) Environments
Source: Cancers (Basel). 2023 Dec 14;15(24):5843. doi: 10.3390/cancers15245843 (PMC10741546; doi:10.3390/cancers15245843)
Supplement: Supplementary file 1 [file cancers-15-05843-s001.zip › Supplementary Table S3A.pptx]

## Slide 1
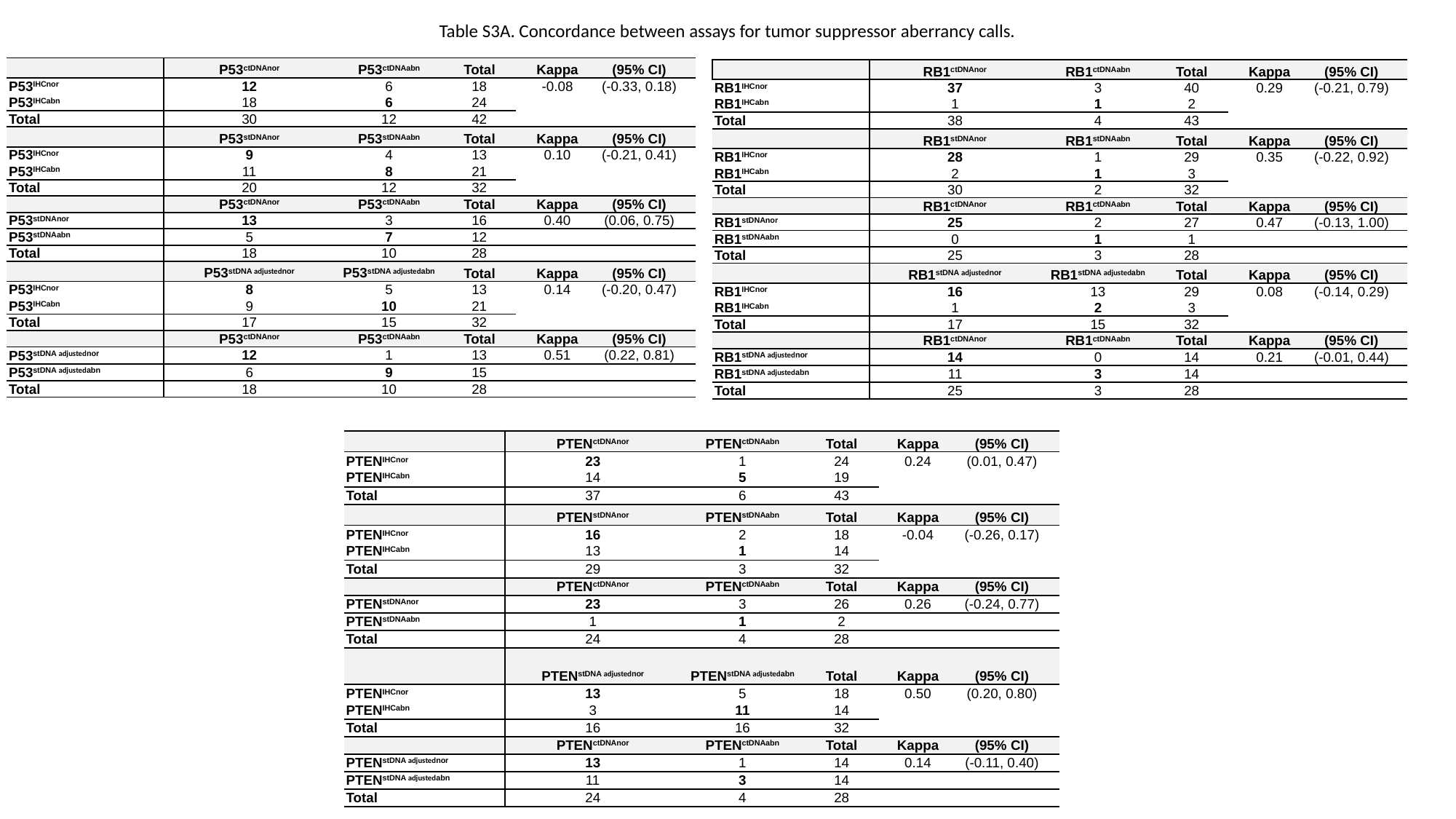

Table S3A. Concordance between assays for tumor suppressor aberrancy calls.
| | P53ctDNAnor | P53ctDNAabn | Total | | Kappa | (95% CI) |
| --- | --- | --- | --- | --- | --- | --- |
| P53IHCnor | 12 | 6 | 18 | | -0.08 | (-0.33, 0.18) |
| P53IHCabn | 18 | 6 | 24 | | | |
| Total | 30 | 12 | 42 | | | |
| | P53stDNAnor | P53stDNAabn | Total | | Kappa | (95% CI) |
| P53IHCnor | 9 | 4 | 13 | | 0.10 | (-0.21, 0.41) |
| P53IHCabn | 11 | 8 | 21 | | | |
| Total | 20 | 12 | 32 | | | |
| | P53ctDNAnor | P53ctDNAabn | Total | | Kappa | (95% CI) |
| P53stDNAnor | 13 | 3 | 16 | | 0.40 | (0.06, 0.75) |
| P53stDNAabn | 5 | 7 | 12 | | | |
| Total | 18 | 10 | 28 | | | |
| | P53stDNA adjustednor | P53stDNA adjustedabn | Total | | Kappa | (95% CI) |
| P53IHCnor | 8 | 5 | 13 | | 0.14 | (-0.20, 0.47) |
| P53IHCabn | 9 | 10 | 21 | | | |
| Total | 17 | 15 | 32 | | | |
| | P53ctDNAnor | P53ctDNAabn | Total | | Kappa | (95% CI) |
| P53stDNA adjustednor | 12 | 1 | 13 | | 0.51 | (0.22, 0.81) |
| P53stDNA adjustedabn | 6 | 9 | 15 | | | |
| Total | 18 | 10 | 28 | | | |
| | RB1ctDNAnor | RB1ctDNAabn | Total | | Kappa | (95% CI) |
| --- | --- | --- | --- | --- | --- | --- |
| RB1IHCnor | 37 | 3 | 40 | | 0.29 | (-0.21, 0.79) |
| RB1IHCabn | 1 | 1 | 2 | | | |
| Total | 38 | 4 | 43 | | | |
| | RB1stDNAnor | RB1stDNAabn | Total | | Kappa | (95% CI) |
| RB1IHCnor | 28 | 1 | 29 | | 0.35 | (-0.22, 0.92) |
| RB1IHCabn | 2 | 1 | 3 | | | |
| Total | 30 | 2 | 32 | | | |
| | RB1ctDNAnor | RB1ctDNAabn | Total | | Kappa | (95% CI) |
| RB1stDNAnor | 25 | 2 | 27 | | 0.47 | (-0.13, 1.00) |
| RB1stDNAabn | 0 | 1 | 1 | | | |
| Total | 25 | 3 | 28 | | | |
| | RB1stDNA adjustednor | RB1stDNA adjustedabn | Total | | Kappa | (95% CI) |
| RB1IHCnor | 16 | 13 | 29 | | 0.08 | (-0.14, 0.29) |
| RB1IHCabn | 1 | 2 | 3 | | | |
| Total | 17 | 15 | 32 | | | |
| | RB1ctDNAnor | RB1ctDNAabn | Total | | Kappa | (95% CI) |
| RB1stDNA adjustednor | 14 | 0 | 14 | | 0.21 | (-0.01, 0.44) |
| RB1stDNA adjustedabn | 11 | 3 | 14 | | | |
| Total | 25 | 3 | 28 | | | |
| | PTENctDNAnor | PTENctDNAabn | Total | | Kappa | (95% CI) |
| --- | --- | --- | --- | --- | --- | --- |
| PTENIHCnor | 23 | 1 | 24 | | 0.24 | (0.01, 0.47) |
| PTENIHCabn | 14 | 5 | 19 | | | |
| Total | 37 | 6 | 43 | | | |
| | PTENstDNAnor | PTENstDNAabn | Total | | Kappa | (95% CI) |
| PTENIHCnor | 16 | 2 | 18 | | -0.04 | (-0.26, 0.17) |
| PTENIHCabn | 13 | 1 | 14 | | | |
| Total | 29 | 3 | 32 | | | |
| | PTENctDNAnor | PTENctDNAabn | Total | | Kappa | (95% CI) |
| PTENstDNAnor | 23 | 3 | 26 | | 0.26 | (-0.24, 0.77) |
| PTENstDNAabn | 1 | 1 | 2 | | | |
| Total | 24 | 4 | 28 | | | |
| | PTENstDNA adjustednor | PTENstDNA adjustedabn | Total | | Kappa | (95% CI) |
| PTENIHCnor | 13 | 5 | 18 | | 0.50 | (0.20, 0.80) |
| PTENIHCabn | 3 | 11 | 14 | | | |
| Total | 16 | 16 | 32 | | | |
| | PTENctDNAnor | PTENctDNAabn | Total | | Kappa | (95% CI) |
| PTENstDNA adjustednor | 13 | 1 | 14 | | 0.14 | (-0.11, 0.40) |
| PTENstDNA adjustedabn | 11 | 3 | 14 | | | |
| Total | 24 | 4 | 28 | | | |
