# Supplementary material for: Evaluation of the Aggressive-Variant Prostate Cancer Molecular Signature in Clinical Laboratory Improvement Amendments (CLIA) Environments
Source: Cancers (Basel). 2023 Dec 14;15(24):5843. doi: 10.3390/cancers15245843 (PMC10741546; doi:10.3390/cancers15245843)
Supplement: Supplementary file 1 [file cancers-15-05843-s001.zip › Supplementary Table S3B.pptx]

## Slide 1
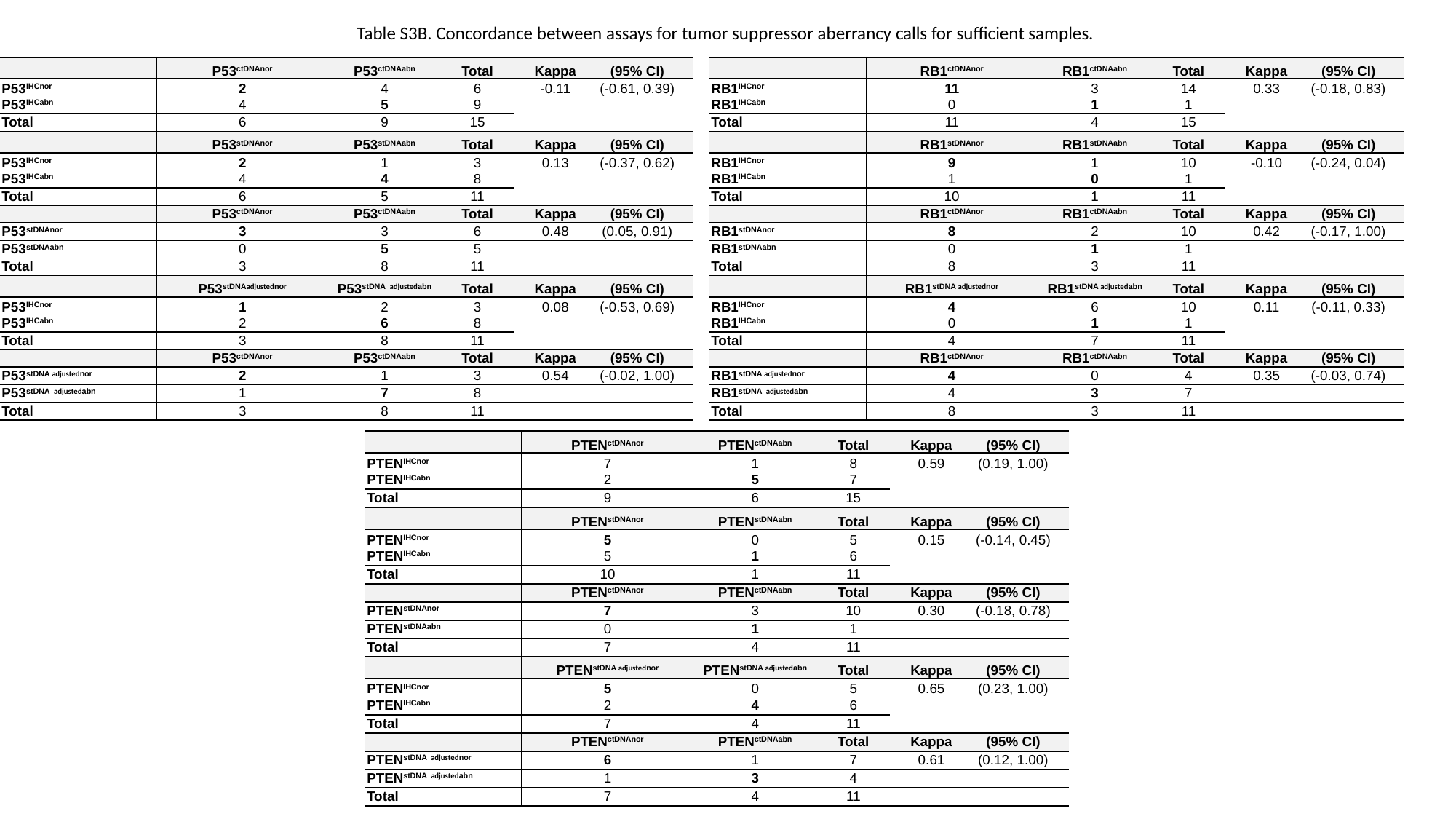

Table S3B. Concordance between assays for tumor suppressor aberrancy calls for sufficient samples.
| | P53ctDNAnor | P53ctDNAabn | Total | | Kappa | (95% CI) |
| --- | --- | --- | --- | --- | --- | --- |
| P53IHCnor | 2 | 4 | 6 | | -0.11 | (-0.61, 0.39) |
| P53IHCabn | 4 | 5 | 9 | | | |
| Total | 6 | 9 | 15 | | | |
| | P53stDNAnor | P53stDNAabn | Total | | Kappa | (95% CI) |
| P53IHCnor | 2 | 1 | 3 | | 0.13 | (-0.37, 0.62) |
| P53IHCabn | 4 | 4 | 8 | | | |
| Total | 6 | 5 | 11 | | | |
| | P53ctDNAnor | P53ctDNAabn | Total | | Kappa | (95% CI) |
| P53stDNAnor | 3 | 3 | 6 | | 0.48 | (0.05, 0.91) |
| P53stDNAabn | 0 | 5 | 5 | | | |
| Total | 3 | 8 | 11 | | | |
| | P53stDNAadjustednor | P53stDNA adjustedabn | Total | | Kappa | (95% CI) |
| P53IHCnor | 1 | 2 | 3 | | 0.08 | (-0.53, 0.69) |
| P53IHCabn | 2 | 6 | 8 | | | |
| Total | 3 | 8 | 11 | | | |
| | P53ctDNAnor | P53ctDNAabn | Total | | Kappa | (95% CI) |
| P53stDNA adjustednor | 2 | 1 | 3 | | 0.54 | (-0.02, 1.00) |
| P53stDNA adjustedabn | 1 | 7 | 8 | | | |
| Total | 3 | 8 | 11 | | | |
| | RB1ctDNAnor | RB1ctDNAabn | Total | | Kappa | (95% CI) |
| --- | --- | --- | --- | --- | --- | --- |
| RB1IHCnor | 11 | 3 | 14 | | 0.33 | (-0.18, 0.83) |
| RB1IHCabn | 0 | 1 | 1 | | | |
| Total | 11 | 4 | 15 | | | |
| | RB1stDNAnor | RB1stDNAabn | Total | | Kappa | (95% CI) |
| RB1IHCnor | 9 | 1 | 10 | | -0.10 | (-0.24, 0.04) |
| RB1IHCabn | 1 | 0 | 1 | | | |
| Total | 10 | 1 | 11 | | | |
| | RB1ctDNAnor | RB1ctDNAabn | Total | | Kappa | (95% CI) |
| RB1stDNAnor | 8 | 2 | 10 | | 0.42 | (-0.17, 1.00) |
| RB1stDNAabn | 0 | 1 | 1 | | | |
| Total | 8 | 3 | 11 | | | |
| | RB1stDNA adjustednor | RB1stDNA adjustedabn | Total | | Kappa | (95% CI) |
| RB1IHCnor | 4 | 6 | 10 | | 0.11 | (-0.11, 0.33) |
| RB1IHCabn | 0 | 1 | 1 | | | |
| Total | 4 | 7 | 11 | | | |
| | RB1ctDNAnor | RB1ctDNAabn | Total | | Kappa | (95% CI) |
| RB1stDNA adjustednor | 4 | 0 | 4 | | 0.35 | (-0.03, 0.74) |
| RB1stDNA adjustedabn | 4 | 3 | 7 | | | |
| Total | 8 | 3 | 11 | | | |
| | PTENctDNAnor | PTENctDNAabn | Total | | Kappa | (95% CI) |
| --- | --- | --- | --- | --- | --- | --- |
| PTENIHCnor | 7 | 1 | 8 | | 0.59 | (0.19, 1.00) |
| PTENIHCabn | 2 | 5 | 7 | | | |
| Total | 9 | 6 | 15 | | | |
| | PTENstDNAnor | PTENstDNAabn | Total | | Kappa | (95% CI) |
| PTENIHCnor | 5 | 0 | 5 | | 0.15 | (-0.14, 0.45) |
| PTENIHCabn | 5 | 1 | 6 | | | |
| Total | 10 | 1 | 11 | | | |
| | PTENctDNAnor | PTENctDNAabn | Total | | Kappa | (95% CI) |
| PTENstDNAnor | 7 | 3 | 10 | | 0.30 | (-0.18, 0.78) |
| PTENstDNAabn | 0 | 1 | 1 | | | |
| Total | 7 | 4 | 11 | | | |
| | PTENstDNA adjustednor | PTENstDNA adjustedabn | Total | | Kappa | (95% CI) |
| PTENIHCnor | 5 | 0 | 5 | | 0.65 | (0.23, 1.00) |
| PTENIHCabn | 2 | 4 | 6 | | | |
| Total | 7 | 4 | 11 | | | |
| | PTENctDNAnor | PTENctDNAabn | Total | | Kappa | (95% CI) |
| PTENstDNA adjustednor | 6 | 1 | 7 | | 0.61 | (0.12, 1.00) |
| PTENstDNA adjustedabn | 1 | 3 | 4 | | | |
| Total | 7 | 4 | 11 | | | |
