# Supplementary material for: Evaluation of the Aggressive-Variant Prostate Cancer Molecular Signature in Clinical Laboratory Improvement Amendments (CLIA) Environments
Source: Cancers (Basel). 2023 Dec 14;15(24):5843. doi: 10.3390/cancers15245843 (PMC10741546; doi:10.3390/cancers15245843)
Supplement: Supplementary file 1 [file cancers-15-05843-s001.zip › Suppplementary Figure S1.pptx]

## Slide 1
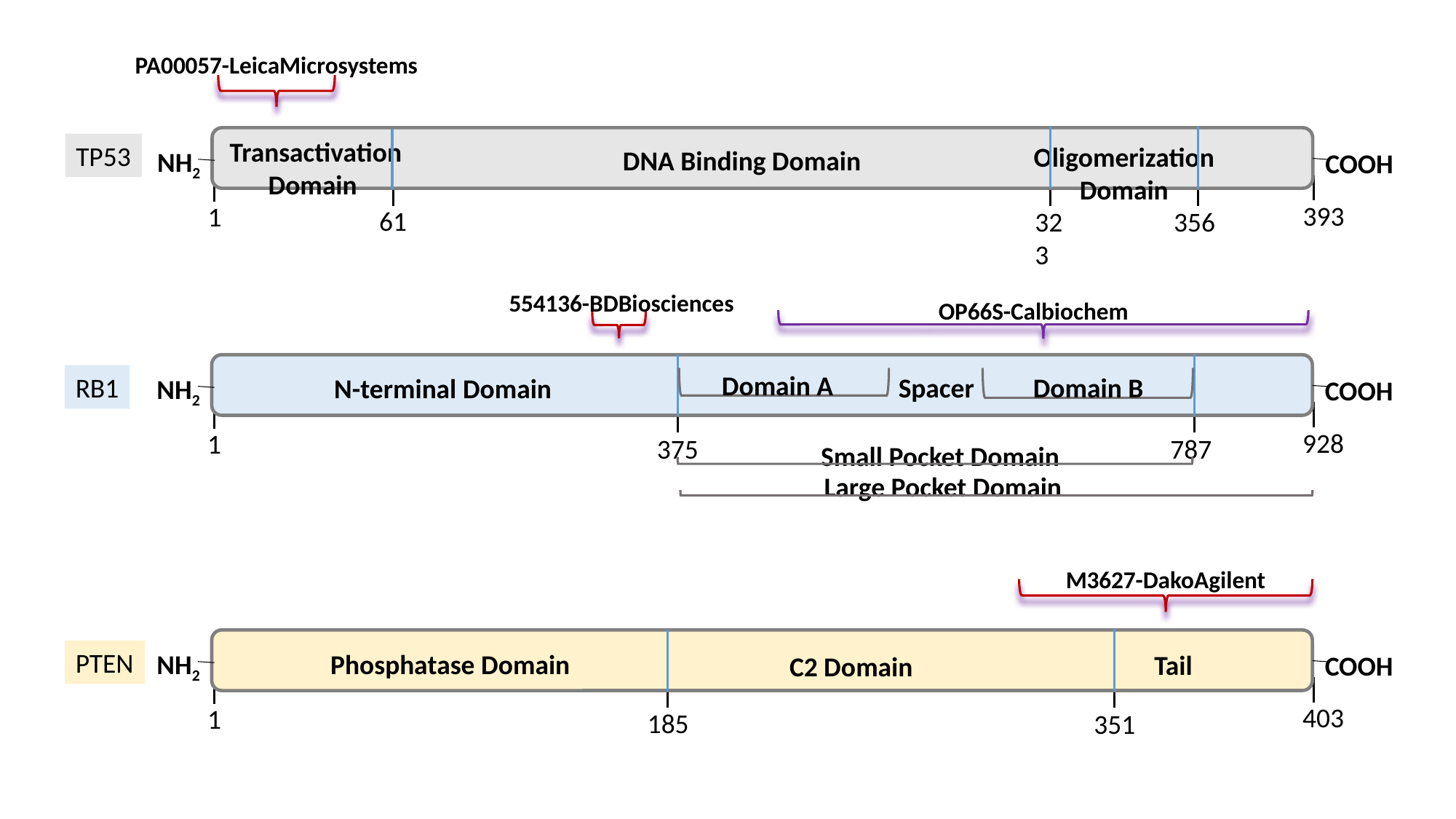

PA00057-LeicaMicrosystems
Transactivation
Domain
TP53
Oligomerization
Domain
DNA Binding Domain
NH2
COOH
393
1
61
323
356
554136-BDBiosciences
OP66S-Calbiochem
Domain A
Spacer
RB1
Domain B
N-terminal Domain
NH2
COOH
928
1
787
375
Small Pocket Domain
Large Pocket Domain
M3627-DakoAgilent
PTEN
NH2
Phosphatase Domain
Tail
COOH
C2 Domain
403
1
185
351
