# Supplementary figures and images for: Evaluation of the Aggressive-Variant Prostate Cancer Molecular Signature in Clinical Laboratory Improvement Amendments (CLIA) Environments
Source: Cancers (Basel). 2023 Dec 14;15(24):5843. doi: 10.3390/cancers15245843 (PMC10741546; doi:10.3390/cancers15245843)

## Slide 1
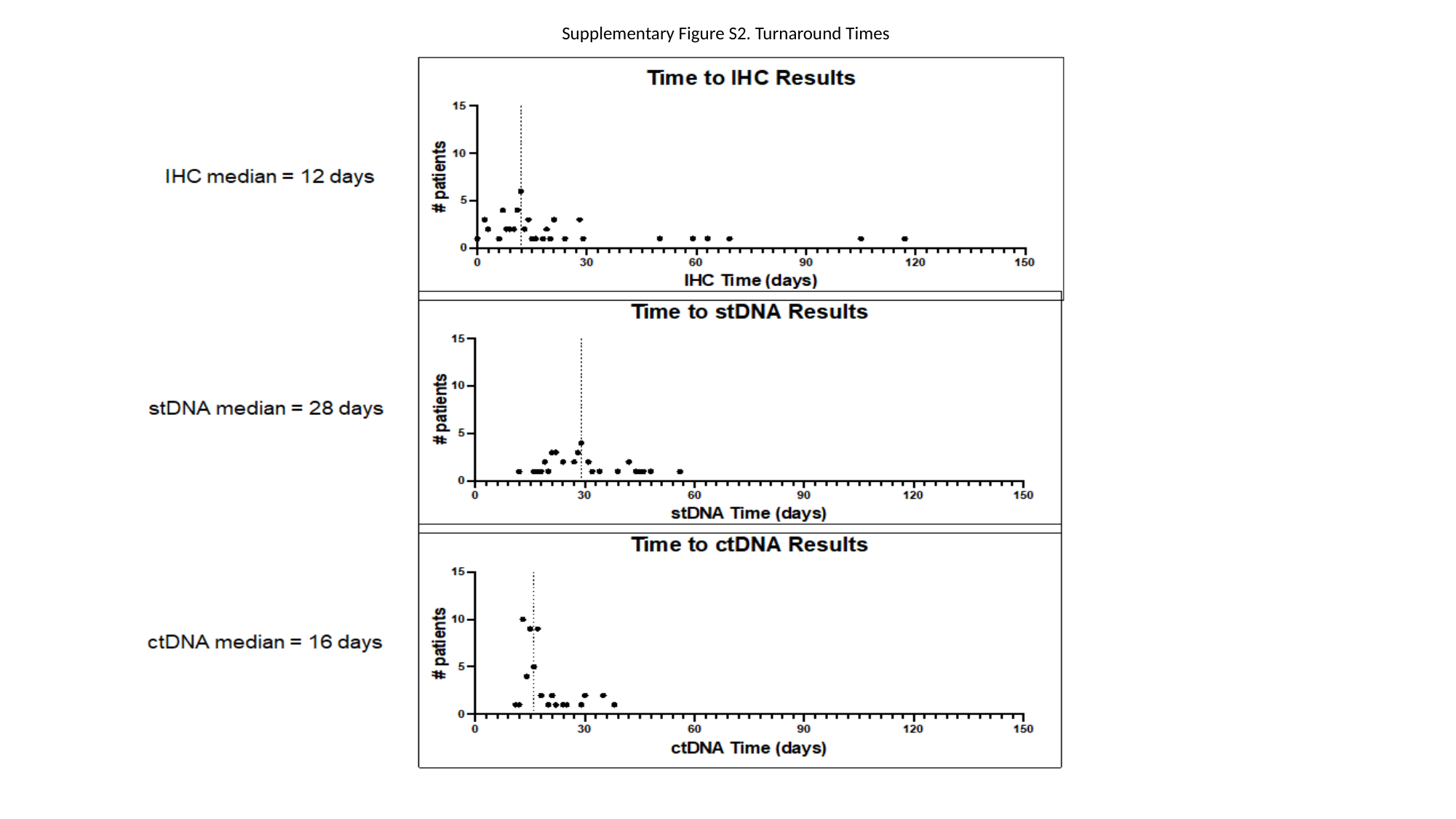

Supplementary Figure S2. Turnaround Times

Supplement: Supplementary file 1 [file cancers-15-05843-s001.zip › Supplementary Figure S2.pptx]
